# Antibiotic-resistant bacteria in the guts of insects feeding on plants: prospects for discovering plant-derived antibiotics

**DOI:** 10.1186/s12866-017-1133-0

**Published:** 2017-12-01

**Authors:** Katarzyna Ignasiak, Anthony Maxwell

**Affiliations:** Department Biological Chemistry, John Innes Centre, Norwich Research Park, Norwich, NR4 7UH UK

**Keywords:** Microbiome, Antibiotic susceptibility, Phytochemicals, Metabolites

## Abstract

**Background:**

Although plants produce many secondary metabolites, currently none of these are commercial antibiotics. Insects feeding on specific plants can harbour bacterial strains resistant to known antibiotics suggesting that compounds in the plant have stimulated resistance development. We sought to determine whether the occurrence of antibiotic-resistant bacteria in insect guts was a widespread phenomenon, and whether this could be used as a part of a strategy to identify antibacterial compounds from plants.

**Results:**

Six insect/plant pairs were selected and the insect gut bacteria were identified and assessed for antibiotic susceptibilities compared with type strains from culture collections. We found that the gut strains could be more or less susceptible to antibiotics than the type strains, or show no differences. Evidence of antibacterial activity was found in the plant extracts from five of the six plants, and, in one case *Catharanthus roseus* (Madagascar Periwinkle), compounds with antibacterial activity were identified.

**Conclusion:**

Bacterial strains isolated from insect guts show a range of susceptibilities to antibiotics suggesting a complex interplay between species in the insect gut microbiome. Extracts from selected plants can show antibacterial activity but it is not easy to isolate and identify the active components. We found that vindoline, present in Madagascar Periwinkle extracts, possessed moderate antibacterial activity. We suggest that plant-derived antibiotics are a realistic possibility given the advances in genomic and metabolomic methodologies.

**Electronic supplementary material:**

The online version of this article (10.1186/s12866-017-1133-0) contains supplementary material, which is available to authorized users.

## Background

Plants are thought to produce over 100,000 secondary metabolites, many of which have antibacterial activity [1]. Phytochemicals have been developed as extremely successful medicines, including the antimalarial drugs quinine [2] and artemisinin [3], and a range of anti-cancer drugs [4], and yet there are no commercially-available plant-derived antibiotics [5].

Plants are a food source for many insect species. The plant-based diet exerts a strong selective pressure on insect-gut bacteria and can potentially lead to antibiotic resistance. In one example, a range of antibiotic-resistance genes was identified in the guts of gypsy moth (*Lymantria dispar*) larvae, feeding on a variety of trees (larch, white oak, willow and aspen) [6]. The cultured gut bacteria were found to be resistant to many common antibiotics: carbenicillin, ceftazidime, chloramphenicol, gentamycin, erythromycin, kanamycin, nalidixic acid, rifampicin, streptomycin, tetracycline and vancomycin. Functional metagenomic analysis of the cultured isolates was conducted to identify novel antibiotic-resistance elements. Libraries of the isolates were prepared as for metagenomic sequencing analyses, but prior to sequencing they were screened by plating on antibiotic-containing media to identify genes conferring resistance to these antibiotics. The analysis revealed three types of genes responsible for the resistant phenotypes: a multidrug resistance protein of the resistance-nodulation-cell division superfamily, an AraC/XylS family transcriptional regulator, and a novel extended-spectrum β-lactamase [6].

These novel resistance elements from *L. dispar* are of clinical relevance. The presence of these genes in insect gut bacteria confers antibiotic-resistance phenotypes and can readily spread to the environments inhabited by the insects [7, 8]. More importantly, the antibiotic-resistance elements present in the insect guts in the absence of clinically-relevant levels of antibiotics suggests that the genes can be maintained in response to other environmental stimuli. We hypothesise that the stimuli responsible for the antibiotic resistance are components in the insect food with antibacterial activity, i.e. these observations suggest that antibiotic compounds are present in the host plant.

A number of insects feed on plants and their products, including plants with medicinal properties. It is not uncommon for insect gut bacteria to be resistant to antimicrobial or toxic components of their foods or to contribute to the detoxification of such compounds. Royal jelly is a honey-bee secretion used for nutrition of queens and larvae and has a potent antimicrobial effect [9]. While core honey bee gut bacteria are resistant to the antibacterial activity of royal jelly, the corresponding environmental strains, non-core species and control strains not associated with bee colonies, are susceptible [10]. In a disc-diffusion assay, six out of eight core isolates were fully resistant to royal jelly, the other two isolates (*Neisseriaceae* sp. and *Lactobacillus* sp. A) were mildly susceptible and the corresponding environmental isolates were fully susceptible. For example, *Lactobacillus kunkeei* gut-isolated strain was fully resistant while the flower-isolated strain was susceptible. It is not clear if such resistance differences in the insect gut microbiota are common; we aimed to explore this question.

To achieve that we selected a number of plants with known medicinal properties, such as eucalyptus, lavender and Madagascar periwinkle, toxic plants, such as ragwort and potato, and a plant with no known medicinal or toxic properties, i.e. cabbage. Each plant was matched with an insect species that could be reared feeding exclusively on that plant. The resulting plant/insect pairs are: Giant Lime Green stick insect (*Diapherodes gigantea*) feeding on Eucalyptus (*Eucalyptus dalrympleana*), Diamondback moth (*Plutella xylostella*) feeding on Chinese Cabbage (*Brassica rapa*), Cinnabar moth (*Tyria jacobaeae*) feeding on Ragwort (*Jacobaea vulgaris*), Rosemary beetle (*Chrysolina americana*) feeding on Lavender (*Lavendula angustifolia*), Death’s-head Hawkmoth (*Acherontia atropos*) feeding on Potato leaves (*Solanum tuberosum*), and Beet Armyworm (*Spodoptera exigua*) feeding on Madagascar Periwinkle (*Catharanthus roseus*). We chose insects feeding exclusively on the selected plant species; to ensure this we either chose monophagous insect species or insects reared in captivity that were feeding exclusively on the selected plant.

The aims of this work were essentially two-fold. Firstly, to see whether the phenomenon of unexpected resistance to antibiotics found in gypsy moth gut bacteria (discussed above) could be found in other insects, i.e. is this a general phenomenon? Secondly, to test whether insect gut bacteria can be utilised in assay-guided fractionation of plant extracts leading to the identification of plant fractions with antibacterial activities. To do this we compared antibiotic-susceptibility profiles and susceptibility to plant extracts of gut-isolated bacterial strains and the corresponding type strains, and used any differences in susceptibilities to identify plant extracts with the most promising antibiotic activity.

## Results

### Giant lime green stick insect feeding on eucalyptus

Three stick insects were analysed in this study and were treated separately, in part to examine the diversity of species from different individuals. From individual 1, three bacterial species were identified: *Bacillus amyloliquefaciens*, *Microbacterium oxydans* and *Sphingobacterium multivorum* (Table 1). *Ba. amyloliquefaciens* and *M. oxydans* are environmental strains, first isolated from soil and air respectively. *Sp. mulitvorum*, on the other hand, was first isolated from the human spleen. As only a small proportion of bacteria can be cultured under laboratory conditions, we decided to assess what proportion of bacterial strains is recovered from the insect gut by culture-dependent methods. To do this, the other two stick insects were dissected and bacteria from their guts were cultivated and identified. Additionally, bacterial genomic DNA (gDNA) was isolated from the samples using a kit optimised for use with soil bacteria and the samples were submitted for 16S sequencing.

**Table 1:** Summary of bacterial species isolated from the guts of various insects feeding on plants

	Phylum			
Insect species	Actinobacteria	Bacteroidetes	Firmicutes	Proteobacteria
Giant Line Green Stick insect (*Diaphroedes gigantea)*	*Microbacterium oxydans*	*Sphingobacterium multivorum*	*Bacillus amyloliquefaciens*	
Diamondback moth (*Plutella xylostella*)	*Sanguibacter keddieii*			*Raoultella terrigena*
Cinnabar moth (*Tyria jacobeae*)	*Kocuria rhizophila*		*Bacillus licheniformis,* *Staphylococcus epidermidis, Staphylococcus warneri*	*Burkholderia fungorum*
Rosemary beetle (*Chrysolina americana*)	*Microbacterium foliorum*, *Microbacterium gubbeenense,* *Rhodococcus erythropolis*		*Staphylococcus epidermidis*	*Pantoea agglomerans*
Death’s-head Hawkmoth (*Acherontia atropos*)				*Enterobacter asburiae,* *Pseudomonas putida, Roultella terrigena*
Beet Armyworm (*Spodoptera exigua*)	*Microbacterium paraoxydans*		*Bacillus aquimaris,* *Bacillus vietnamensis*	*Rhizobium pusense*

The two samples, originating from two separate stick insects, contained 9450 and 8450 valid reads, yielding 97 and 53 operational taxonomic units (OTUs) respectively. The rarefaction curve reached a plateau indicating that the bacterial diversity in the guts has been sufficiently sampled (Additional file 1: Figure S1). Out of the sample with 97 different OTUs, only 12 were present at above 1% abundance; the most abundant strain was *Serratia marcescens*, which constituted 33% bacterial reads retrieved from the *D. gigantea* guts (Additional file 2: Figure S2). The other gut sample contained 53 OTUs; five of these were present above 1% abundance. Again, *Se. marcescens* was the dominating strain in the gut community with 91% abundance. *Se. marcescens* is a common pathogen of insects but it can be also found in healthy insects. The only bacterial order found in the guts was Enterobacteriales. Other strains shared by the two gut communities are: *Pantoea beijingensis*, *Lellottia amnigena*, and *Enterobacter asburiae* (Additional file 2: Figure S2).

Because the metagenomic analysis was performed on a new set of insect guts, we cultured and identified strains from these samples as described before. A set of strains was identified: *Serratia marcescens*, *Microbacterium paraoxydans*, *Kocuria rhizophila* and *Pectobacterium cypripedi*. The older samples, used for the assessment of antibiotic resistance in the insect gut, contained a different set of bacterial strains (see above). It is perhaps surprising that no strains are shared between the two sets of *D. gigantea* guts; the only closely related strains are the two *Microbacterium* species. This may reflect intrinsic differences between the gut microbiota between individuals and/or experimental variations such that different bacterial species were dominant in the plated samples. Notwithstanding this, our comparison of culture-dependent and –independent methods suggest that we can culture <5% strains from the *D. gigantea* guts.

For the antibiotic susceptibility trials, the 3 bacterial species isolated from individual 1 were analysed. In most cases there was no difference in antibiotic susceptibility between the type strains and gut-isolated strains (Table 2). *Ba. amyloliquefaciens* gut-isolated strain was more resistant to ciprofloxacin and tetracycline than the type strain. *M. oxydans* from the stick insect gut was more resistant to ciprofloxacin, but less resistant to tetracycline when compared to the type strain. Gut-isolated *Sp. multivorum* was more susceptible to tetracycline than the type strain, perhaps unsurprisingly as the type strain is a clinical isolate.

**Table 2:**
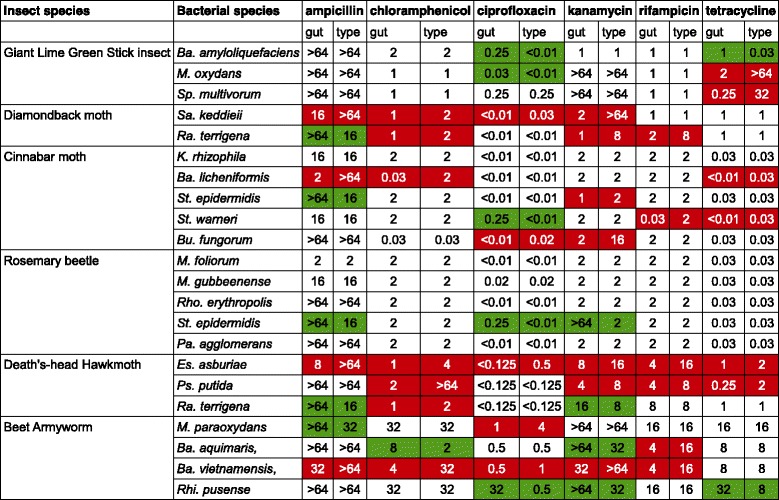
Antibiotic susceptibility profiles of the bacteria isolated from the insect guts. The antibiotic susceptibility of the gut-isolated strains and the type strains was assessed in a broth microdilution assay (green indicates gut strain less susceptible than type strain; red indicates gut strain more susceptible than type strain)

We assessed the extract from eucalyptus leaves for its ability to inhibit the growth of the three bacterial species isolated from the gut of individual number 1 (Table 3). No differences in plant extract susceptibility were observed for *Ba. amyloliquefaciens*; both the gut-isolated strain and type strain were fully resistant to the eucalyptus extract. The gut-isolated *Sp. multivorum* strain was more resistant to the eucalyptus extract than the type strain. Surprisingly, the opposite was observed for *M. oxydans*; the gut-isolated strain was more susceptible to eucalyptus extract than the type strain. *M. oxydans* has shown unexpected ciprofloxacin resistance in the antibiotic susceptibility testing and it is possible that the strain is generally more resistant to several compounds or conditions.

**Table 3:** The susceptibility of the bacteria isolated from insect guts and corresponding type strains to the plant extracts

Insect species	Food plant	Bacterial species	Zone of inhibition [mm]							
			Crude leaf		Crude flower		Crude root		HPLC fraction	
			gut	type	gut	type	gut	type	gut	type
Giant Lime Green Stick insect (*Diaphroedes gigantea)*	Eucalyptus (*Eucalyptus dalyrympleana*)	*Ba. amyloliquefaciens*	0	0					0	0
		*M. oxydans*	7	4					12	10
		*Sp. multivorum*	10	17					7	14
Diamondback moth (*Plutella xylostella*)	Chinese Cabbage (*Brassica rapa*)	*Sa. keddieii*	0	0						
		*Ra. terrigena*	0	0						
Cinnabar moth (*Tyria jacobeae*)	Ragwort (*Jacobaea vulgaris*)	*K. rhizophila*	23	25						
		*Ba. licheniformis*	27	27						
		*St. epidermidis*	23	21						
		*St. warneri*	18	16						
		*Bu. fungorum*	23	18						
Rosemary beetle (*Chrysolina americana*)	Lavender (*Lavendula augustifolia*)	*M. foliorum*	7	6	10	10				
		*M. gubbeenense*	6	6	11	10				
		*Rho. erythropolis*	0	0	10	10				
		*St. epidermidis*	0	0	10	12				
		*Pa. agglomerans*	0	0	12	12				
Death’s-head Hawkmoth (*Acherontia atropos*)	Potato (*Solanum tuberosum*)	*E. asburiae*	7	7						
		*Ps. putida*	6	6						
		*Ra. terrigena*	10	7						
Beet Armyworm (*Spodoptera exigua*)	Madagascar Periwinkle (*Catharansus roseus*)	*M. paraoxydans*	8	6			28	20		
		*Ba. aquimaris,*	6	6			16	0		
		*Ba. vietnamensis*,	6	6			17	0		
		*Rhi. pusense*	6	7			17	20		

The values were obtained in a disc diffusion assay; the discs carried 10 μg dried extract, the assay was repeated three times

After we established that the eucalyptus extract has antibacterial activity, we attempted to purify an active fraction of the extract (see Methods). After initial purification, HPLC fractions were tested for antibacterial activity and fractions with antibacterial activity was subjected to another round of HPLC, but further separation of the compounds was not achieved. Liquid chromatography coupled with mass spectrometry (LC-MS) analysis of the HPLC-purified fraction revealed the presence of a complex mixture of compounds. Considering only the most abundant ions, positive electrospray MS revealed between 20 and 25 different molecular ions, ranging between m/z 264.8250 and 630.9556. Further isolation of individual components from this mixture and elucidation of their chemical structures were not attempted.

### Diamondback moth feeding on Chinese cabbage

The strains identified from *Plutella xylostella* guts were *Sanguibacter keddieii* and *Raoultella terrigena* (Table 1). *Sa. keddieii* is a rare bacterial species, previously isolated from the blood of otherwise healthy cows [11]. *Ra. terrigena* was previously classified as *Klebsiella terrigena* and can be isolated from soil and water [12]. In antibiotic susceptibility trials (Table 2), the *Ra. terrigena* type strain was less resistant to ampicillin than the gut-isolated strain and more resistant to chloramphenicol, kanamycin and rifampicin. The *Sa. keddieii* type strain was more resistant to ampicillin, chloramphenicol, ciprofloxacin and kanamycin than the gut-isolated strain. The differences in antibiotic resistance levels of *Sa. keddieii* were unusual and higher than the other strains tested. We found that the cabbage extract did not inhibit the growth of either of the *P. xylostella* gut or type strains (Table 3). Since only two strains were isolated from the guts of Diamondback moth larvae, we tested additional strains: an indicator strain *Escherichia coli* ATCC 25922, and strains isolated from the stick insect gut: *Ba. amyloliquefaciens*, *M. oxydans* and *Sp. multivorum*, with their corresponding type strains. No antibacterial activity was observed against any of these strains (results not shown). No further work was carried out on the cabbage leaf extract.

### Cinnabar moth feeding on ragwort

Five bacterial strains were isolated from the guts of cinnabar moth larvae feeding on ragwort leaves (Table 1): *Burkholderia fungorum*, *Bacillus licheniformis*, *Kocuria rhizophila*, *Staphylococcus epidermidis* and *Staphylococcus warneri*. *Staphylococcus* species are common members of human skin microbiota [13], but it is not uncommon for insect gut microbiota to include such isolates. It is unlikely these species are a contamination introduced during gut dissections as the procedure was carried out in sterile conditions following appropriate dissection procedures. *St. epidermidis* and *St. warneri* are the only isolates in the guts of cinnabar moth larvae that are normally associated with human microbiota. All the remaining bacterial isolates are environmental strains: *Bu. fungorum* was first isolated from a fungus and both *Ba. licheniformis* and *K. rhizophila* are soil bacteria. In antibiotic susceptibility trials (Table 2), the type strains were often more resistant to antibiotics. The *Bu. fungorum* type strain was more resistant than the gut-isolated strain to ciprofloxacin and kanamycin. The *Ba. licheniformis* type strain was more resistant to three antibiotics than the gut-isolated *Ba. licheniformis*: ampicillin, chloramphenicol and oxytetracycline. There were no differences in antibiotic susceptibility between *K. rhizophila* gut-isolated strain and the type strain. The *St. epidermidis* gut-isolated strain was more resistant to ampicillin and less resistant to kanamycin than the type strain and the *St. warneri* gut-isolated strain was more resistant to ciprofloxacin than the type strain. Additionally, the *St. warneri* type strain was more resistant to rifampicin and tetracycline than the gut strain. The large number of cases where the type strain was more antibiotic-resistant than the gut-isolated strain was unexpected.

There were no differences in ragwort extract susceptibility between the gut-isolated strain and type strain of *Ba. licheniformis* (Table 3). Four gut-isolated strains were more resistant to the ragwort extract than the corresponding type strains: *Bu. fungorum*, *St. epidermidis* and *St. warnerii*. *K. rhizophila* gut-isolated strain was more susceptible to the ragwort extract than the type strain. Surprisingly, the strains were equally antibiotic-susceptible, making it difficult to hypothesise what caused increased resistance to the plant extract.

Even though three out of five type strains tested were more resistant to the plant extract than their corresponding gut strain, the ragwort extract exhibited a high level of antibiotic activity and the extract was investigated further. The partitioned ragwort extract was fractionated on a weak anion exchange SPE column followed by reverse-phase HPLC. Fractions exhibiting antibacterial activity were further analysed by LC-MS. Known pyrrolizidine alkaloids and their N-oxides, which are the compounds responsible for the toxicity of ragwort to cattle [14], were not detected. These alkaloids include: jacobine (m/z 351), jaconine (m/z 387), jacozine (m/z 349), otosenine (m/z 381), retrorsine (m/z 351), seneciphilline (m/z 333), senecionine (m/z 335), and senkirkine (m/z 366), acetylerucifoline (m/z 392), erucifoline (m/z 350), integerrimine (m/z 335), jacoline (m/z 369), riddelline (m/z 350), senecivernine (m/z 335), spartioidine (m/z 333), and usaramine (m/z 351). The N-oxides have m/z ratios 16 mass units higher than the parent alkaloids. The absence of these metabolites in our extracts suggests that the pyrrolizidine alkaloids were either not extracted from the plant material or not selected for in the assay-guided HPLC fractionation, and are not responsible for the antibacterial activity of the ragwort extract. However, the generally high level of activity of the ragwort extract suggests that this warrants future investigation.

### Rosemary beetle feeding on lavender

We were not able to dissect the guts of rosemary beetles and so instead we isolated total bacteria from surface-sterilized beetles. Five different bacterial strains were isolated: *Microbacterium foliorum*, *Microbacterium gubbeenense*, *Pantoea agglomerans*, *Rhodococcus erythropolis* and *Staphylococcus epidermidis*. The Rosemary beetle microbiota is probably dominated by the gut bacteria; three of the isolated strains were of environmental origin: *M. foliorum* was first isolated from the surface of leaves, *M. gubbeenense* from cheese and *Rho. erythrypolis* from soil. The two remaining bacterial strains were human-associated: both *Pa. agglomerans* and *St. epidemidis* can be found on human skin.

In comparison with other insect gut microbiomes there were few differences in antibiotic susceptibilities between the strains isolated from the Rosemary beetles and the matching type strains (Table 2). There were no differences in antibiotic susceptibility to ampicillin, chloramphenicol, ciprofloxacin, kanamycin, rifampicin or oxytetracycline between the type strains and insect-isolated strains of *M. foliorum*, *M. gubbeenense*, *Pa. agglomerans* and *Rho. erythropolis*. The *St. epidermidis* type strain was more susceptible to ampicillin, ciprofloxacin, and kanamycin than the gut strain. Even though not many differences in the antibiotic susceptibility between the strains have been detected, in three cases when this was the case the insect-isolated strain was more resistant than the type strain, indicating it was under selective pressure.

Rosemary beetles feed on lavender leaves, but the plants harvested for the preparation of the lavender extract also had flowers. For the purposes of comparison, leaf and flower lavender extracts were prepared and assayed for antibiotic properties. The leaf extract was antibacterial against two of the bacterial strains used and the flower extract was active against all five strains. Both extracts exhibited only a low level of activity. In most cases there were no differences in the susceptibility to lavender extracts between the type and the ‘gut’ strains. The *St. epidermidis* insect strain was more resistant to lavender flower extract than the type strain and the *M. foliorum* type strain was more resistant to lavender leaf extract than the insect-isolated strain.

Generally, only a low level of antibacterial activity was detected in lavender and the flower extract tended to be more active than the leaf extract. Only the flower extract was investigated further due to its higher antibacterial activity. The lavender flower extract was subjected to purification with SPE and HPLC. Low-level antibacterial activity was detected in five HPLC fractions ranging between 17 and 50% methanol. The low level of activity hindered the assay-guided fractionation as separating the peaks by HPLC lowered the individual antibiotic activity of each peak below detection level. Semi-preparative HPLC separation did not provide sufficient amounts of material to allow identification of the most active components of the extract.

### Death’s-head hawkmoth feeding on potato leaves

Three different bacterial strains were isolated from the guts of Death’s-head Hawkmoths: *Enterobacter asburiae*, *Pseudomonas putida* and *Raoultella terrigena*. *En. asburiae* is a species normally isolated from clinical specimens while the two other isolates, *P. putida* and *Ra. terrigena*, are environmental bacteria commonly isolated from soil and water.

For Death’s-head Hawkmoth bacteria there were differences in antibiotic susceptibility in the majority of tests performed and most of the type strains were more antibiotic-resistant than their matching gut-isolated strain (Table 2). The *En. asburiae* type strain was more resistant than the gut-isolated strain to all antibiotics tested: ampicillin, chloramphenicol, ciprofloxacin, kanamycin, rifampicin and tetracycline. The *Ps. putida* type strain was more resistant to chloramphenicol, kanamycin, rifampicin and tetracycline. *Ra. terrigena* type strain was more resistant than the gut strain to chloramphenicol and more susceptible than the gut strain to kanamycin and tetracycline.

There were no differences in potato leaf extract susceptibility between the gut isolates and type strains of *En. asburiae* and *Ps. putida* (Table 3). The *Ra. terrigena* gut-isolated strain was more susceptible to the potato extract than the type strain, contrary to what was expected. The strains had some unexpected antibiotic resistance differences. The type strain was more chloramphenicol-resistant than the gut-isolated strain, opposite to the predicted resistance profile. It is possible that both the chloramphenicol resistance and the increased potato extract resistance are due to the same mechanism. As the antibacterial activity of the extract fractions were relatively low this was not further pursued.

### Beet armyworm feeding on Madagascar periwinkle

Four bacterial strains were isolated from Beet Armyworm guts: *Bacillus aquimaris*, *Bacillus vietnamensis*, *Microbacterium paraoxydans* and *Rhizobium pusense*. *Ba. aquamaris*, *Ba. vietnamensis* and *Rhi. pusense* are environmental isolates, and *M. paraoxydans* is a clinical isolate. As discussed before it is not unusual for an insect gut to contain both environmental strains and isolates of clinical importance.

Differences in antibiotic susceptibility were detected in over half of the tests performed. The *Ba. aquimaris* type strain was more susceptible to chloramphenicol and kanamycin, but more resistant to rifampicin than the gut-isolated strain. The *Ba. vietnamensis* type strain was more resistant to five out of six antibiotics tested: ampicillin, chloramphenicol, ciprofloxacin, kanamycin and rifampicin. The *M. paraoxydans* type strain was more susceptible to ampicillin and more resistant to ciprofloxacin than the gut-isolated strain. The *Rhi. pusense* gut-isolated strain was more resistant than the type strain to ciprofloxacin, kanamycin and tetracycline.

Both leaf and root extracts were prepared from the Madagascar Periwinkle (*C. roseus*) plants. There were no differences in Madagascar Periwinkle leaf extract susceptibility between the gut strains and type strains of the two *Bacillus* isolates: *Ba. aquamaris* and *Ba. vietnamensis*. The *Rhi. pusense* gut isolate was more resistant than the type strain to both the leaf and root extract. Contrary to what was expected, in several cases the type strain was more resistant to the *C. roseus* extract than the gut-isolated strain. The *M. paraoxydans* type strain was more resistant to both the leaf and root extract than the gut strain and both *Ba. aquamaris* and *Ba. vietnamensis* type strains were fully resistant to the *C. roseus* extract while the gut-isolated strains were susceptible. All three type strains that were unusually resistant to the plant extract, also showed unexpected resistance to antibiotics (Table 2).

**Fig. 1:**
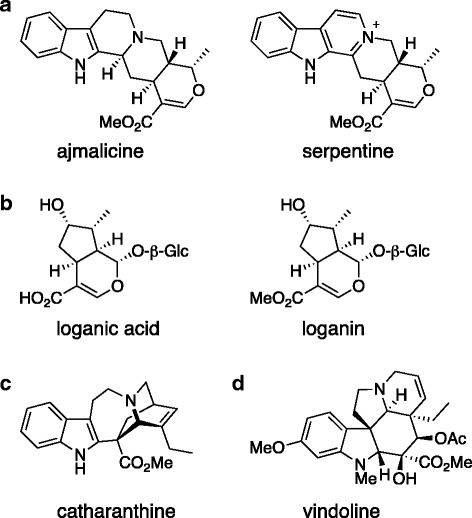
Chemical structures of the identified metabolites from *C. roseus* extract with antibacterial activity. The active fraction from the root extract contained serpentine (**a**), loganic acid (**b**) and catharanthine (**c**); the leaf sample contained vindoline (**d**) as the most abundant metabolite, followed by loganic acid (**b**) and serpentine (**a**). For the experiments in Table 6, ajmalicine (**a**) and loganin (**b**) were used as these are commercially available. Glc = glucose

The Madagascar Periwinkle root extract was purified and fractionated. The active fraction eluted at the beginning of the methanol gradient with fractions containing less than 15% methanol, indicating high polarity of the active components. The HPLC fraction with antibiotic activity was analysed by LC-MS to determine which metabolites were present. The most abundant ions were identified by running their m/z ratios against the annotated metabolites in the Medicinal Plants Consortium database (http://metnetdb.org/mpmr_public/).The most abundant ion (m/z 349.1700) detected was serpentine, a known terpenoid indole alkaloid previously isolated from roots of the Madagascar Periwinkle [15]. Present in the root extract were also loganic acid and catharanthine. The structures of the metabolites with known structures are shown in Fig. 1. The other abundant ions were matched to metabolites with unknown structures. The identifiers of these metabolites are shown in Table 4, but the database does not provide any structural information about these compounds.

**Table 4:** The most abundant ions present in the periwinkle root extract fraction with antibacterial activity, detected by LCMS-IT-ToF Mass Spectrometer (IT-ToF, Shimadzu) using analytical C18 column and 10–100% acetonitrile gradient against 0.1% formic acid in water

Ion m/z	Retention time [min.]	Compound name
349.1700	7.755	serpentine
397.2112	6.928	MDC-Cat-HPLC-E-POS-F1–397.2–6.93
315.0734	6.429	MDC-Cat-HPLC-E-POS-F1–315.119-6.43
396.2036	10.808	MDC-Cat-HPLC-E-POS-F1–396.184-10.81
350.1733	5.658	MDC-Cat-HPLC-E-POS-F1–350.172-5.66
377.0871	2.360	loganic acid
349.1539	2.893	serpentine
337.1836	7.153	catharanthine
398.2159	10.314	MDC-Cat-HPLC-E-POS-F1–398.145-10.31
397.2108	6.928	MDC-Cat-HPLC-E-POS-F1–397.2–6.93

The ions were matched with known metabolites from the *C. roseus* metabolomics database based on their m/z ratios. For ions without a good match in the database, the closest match was listed

The most abundant ion identified in the Madagascar Periwinkle leaf extract was vindoline, followed by loganic acid, and serpentine (Table 5). As before, several ions were matched to metabolites with an unknown structure. Some of the metabolites were shared between the leaf and root extracts. Both serpentine and loganic acid were found in the root extract and leaf extract.

**Table 5:** Most abundant ions present in the *C. roseus* leaf extract. A list of most abundant ions present in the periwinkle leaf extract, detected by LCMS-IT-ToF Mass Spectrometer (IT-ToF, Shimadzu) using analytical C18 column and 10–100% acetonitrile gradient against 0.1% formic acid in water

Ion m/z	Retention time [min.]	Compound name
457.2317	0.829	vindoline
397.2108	2.193	MDC-Cat-HPLC-E-POS-F1–397.2–6.93
377.0871	2.360	loganic acid
349.1539	2.893	serpentine
521.06	1.884	MDC-Cat-HPLC-E-POS-F1–521.204-2.05
458.2358	0.829	MDC-Cat-HPLC-E-POS-F1–458.207-9.83
439.219	0.84	MDC-Cat-HPLC-E-POS-F1–439.202-9.5
520.9128	0.400	MDC-Cat-HPLC-E-POS-F1–520.217-9.48
656.8851	0.200	MDC-Cat-HPLC-E-POS-F1–656.345-13.73
397.2112	5.554	MDC-Cat-HPLC-E-POS-F1–397.2–6.93

The ions were matched with some known metabolites from the *C. roseus* metabolomics database based on their m/z ratios. For ions without a good match in the database, the closest match was listed

Neither of the fractions contained a single metabolite and it is difficult to hypothesise which one of the metabolites identified was responsible for the antibiotic activity. Instead we established minimal inhibitory concentrations (MICs) for commercially-available compounds detected in the fractions (Table 6). In some cases, we used chemically similar compounds that were commercially available; the differences between the metabolites from the periwinkle extracts and tested compounds are shown in Fig. 1 (a, b). Vindoline had the lowest MIC (150–600 μg/mL); previous data also showed vindoline to be the most active terpenoid indole alkaloid, but the MIC was found to be 1000 μg/mL [16]. The other compounds tested, ajmalicine, catharanthine and loganin, had MICs between 300 and 1250 μg/mL. The MIC values found were relatively high; the lowest MIC values were obtained for permeable *Es. coli* strain, consistent with more effective entry into the bacteria.

**Table 6:** Minimal inhibitory concentrations of the main metabolites from *C. roseus* extracts. Where the metabolite was not commercially available we tested the closest metabolite or precursor that was available

Bacterial strain	Minimal inhibitory concentration [mg/mL]			
	vindoline	loganin	ajmalicine	catharanthine
*Escherichia coli*	0.3	0.6	0.6	0.6
*Escherichia coli* (permeable)	0.15	0.3	0.6	0.3
*Mycobacterium smegmatis*	0.6	0.6	0.6	0.6
*Pseudomonas aeruginosa*	0.3	0.6	0.6	0.6
*Staphylococcus aureus*	0.3	0.6	0.6	0.6
*Bacillus vietnamensis*	0.3	0.6	0.6	1.25
*Microbacterium paraoxydans*	0.3	0.6	0.6	0.6
*Rhizobium pusense*	0.3	0.6	0.6	0.6

## Discussion

### Diversity of bacterial strains present in the insect guts

We isolated a variety of bacterial strains from the guts of the insects investigated. Most of the bacterial isolates were common soil and water strains, but we also identified several strains with clinical significance in human health. Such composition of insect gut microbiomes is not unusual and has been described before (for example *Staphylococcus* species in termites [17] and *Enterococcus* species in *Galleria mellonella* (greater wax moths) [18]). As insects are sometimes vectors of human disease, the presence of pathogens in the healthy insect microbiota is an important factor to bear in mind.

We did not identify any obligatorily anaerobic bacteria from the insect guts; however, this may well be a consequence of our methods of isolation. Oxygen levels in herbivorous insects’ guts are low [19], especially in the midgut and hindgut. The oxygen level depends partially on the insect food and increases when the insects feed on artificial food. The presence of oxygen in the guts of herbivorous insects can lead to the formation of reactive oxygen species from the ingested plant material. Additionally, the autoxidation of plant phenolics is greatly reduced by the lack of oxygen, even in the highly alkaline guts of some insects. The depletion of oxygen from the insect guts can be abolished by boiling, suggesting either endogenous insect enzymes or microbial activity are responsible. It is possible that both processes are involved and both the host insect and the native microbiota together sustain the gut as a suitable niche.

The metagenomic analysis of the Giant Lime Green Stick insect guts suggested that we are only able to identify a minority of bacterial species (<5%) by culture-dependent methods. This is not surprising given previous findings [20, 21], but assessing the complete gut microbiome of insects was not one of the aims of this study. The metagenomic analysis did reveal some anaerobic species, but our methods may have biased the results in favour of aerobes.

### Comparison of the gut bacteria isolated in our study with other studies

For the giant stick insects there were few bacterial species shared between our study and previous findings [22, 23]. Diamondback moth and Beet Armyworm microbiomes shared little similarity between our study and previous metagenomic surveys [24, 25]. For other insects there were no studies that we could compare our results to, but the bacterial species we isolated were not uncommon in other insect guts.

A study focusing on the characterisation of order Phasmatodea (stick insects) gut anatomy and microbiology implemented culture-dependent methods to find four microbial species in the gut of *D. gigantea* feeding on *Eucalyptus citriodora* [22]. In the crop there was one bacterial and one fungal species: *Serratia marcescens* and *Cryptococcus ramirezgomezianus,* the midgut was colonised exclusively by *Enterobacter cloacea*, and the hindgut was the most species-rich with three bacterial strains: *En. cloacea*, *Erwinia persicina*, *Se. marcescens*. Another study identified *Se. marcescens* and Spiroplasma species in *D. gigantea* guts via targeted culture-independent methods [23]. The authors did not speculate about the role of *Se. marcescens*, but Spiroplasma species are a common commensal endosymbiont, maternally transmitted, and in some insects responsible for impaired reproduction by inducing the selective elimination of male progeny. Our metagenomic analysis of the stick insect gut community confirms the abundance of *Se. marcescens* in the digestive tract. However from genomic data alone it is not possible to propose a role for *Se. marcescens*, which is both a human pathogen, common in hospital-acquired infections [26], and an insect pathogen sometimes utilized as a model species in immunity studies [27].

On the other hand there was little similarity between the bacterial species isolated from the Diamondback moth larvae and Beet Armyworm larvae in our experiments and previously reported studies [24, 25]. Only two strains have been isolated from the diamondback moth larvae by culture-dependent methods: *Sa. keddieii* and *Ra. terrigena* in our experiments. A previous study identified on average 208 operational taxonomic units from seven *P. xyllostella* larvae midguts [25]. Surprisingly only 25 operational taxonomic units had frequency higher than 0.05%, demonstrating that the majority of isolates present in the guts of Diamondback moth larvae were extremely rare. The authors do not discuss the composition of the *P. xylostella* microbiomes on the species level, but their dataset can be retrieved through Sequence Read Archive (http://www.ncbi.nlm.nih.gov/Traces/sra/).An analysis of the dataset revealed no sequences with high degree of similarity to either *Sa. keddieii* or *Ra. terrigena*, but the rarefaction curves of the dataset did not reach a plateau, indicating that the sequencing depth was insufficient to discover all sequences present in each sample. Another possible explanation is that the strains are not present at all, since the Diamondback moth microbiota may be variable or transient.

Previous studies identified *Ochrobactrum* sp. and *Myroides odoratus* as the main culturable strains from Beet Armyworm guts [24]. *Rhizobium pusense*, isolated in our experiments, is related to *Ochrobactrum* strains. It is possible that these strains are consistently present in the beet armyworm guts. The Beet Armyworm microbiota was shown to be involved in the production of N-acylaminoacids [24], which can be isolated from insects’ oral secretions and have been indicated in eliciting plant defences. One of such N-acylamino acids, microbially-produced volicitin (N-(17-hydroxylinolenoyl)-L-glutamine) induces damaged leaves to produce volatile compounds attracting parasitic wasps that infect the armyworm larvae [28].

For cinnabar moth larvae, rosemary beetle, and Death’s-head Hawkmoth larvae there are no studies available for comparison. Out of five species of bacteria isolated from cinnabar moth guts, two belonged to genus *Staphylococcus*: *St. epidermidis* and *St. warneri*. Both of these isolates are typical members of human skin microbiota. It is not uncommon for bacteria considered typically human to be found in insects. *Staphylococcus* species have been described as members of the gut microbiota of Termitidae family of termites and the presence of *Staphylococcus* species was specific to that group of Australian higher termites [17]; the role of the *Staphylococcus* species in the termite gut is probably cellulose degradation [29].

The rosemary beetle microbiome was another example of an insect gut community with members typically associated with human microbiota: *St. epidermidis* and *Pa. agglomerans*. As discussed before it is not uncommon for insects to harbour such bacteria in their guts. *Pa. agglomerans* is a prominent member of the desert locust *Schistocerca gregaria* [30] and produces compounds that lead to the swarming behaviour of the locusts and are necessary for the synthesis of an aggregation/cohesion pheromone.

The Death’s-head Hawkmoth gut community was a mixture of environmental strains and strains associated with human microbiota, similar to other insect species described in this chapter. Surprisingly, the Death’s-head Hawkmoth larvae harbored strains that were most different from the type strains when the antibiotic susceptibility profiles were compared. The *En. asburiae* type strain is a clinical isolate and it was the only type strain tested that was more resistant than the gut strain to every antibiotic in the panel. This result suggests the Death’s-head Hawkmoths’ gut is not a niche with an extremely selective environment, as the gut isolated *En. asburiae* was not as antibiotic-resistant as the type strain.

### Antibiotic-resistant bacteria can be present in the guts of insects feeding on plants

When the antibiotic-resistance profiles of the gut-isolated strains and the type strains are compared, there are three possible outcomes. Firstly, no differences between the antibiotic resistance levels can be detected. Secondly, the gut-isolated strain can be more resistant than the type strain, and finally, the type strain can be more antibiotic-resistant than the gut strain. Initially it was hypothesised that no differences and higher resistance of the gut-isolated strains would be the most common outcomes of the antibiotic susceptibility testing, i.e. we expected the gut strains to have developed antibiotic resistance in response to selective pressures in the insect gut. We found that equally often as there being no difference in antibiotic resistance or the gut-isolated strain being more antibiotic-resistant, the type strain had a higher level of resistance to the antibiotics tested (Table 2). There are several possible explanations for this phenomenon. For example, it is possible that the elevated antibiotic resistance of some type strains is due to their origin as clinical isolates. Bacteria first cultured in hospital settings are likely to have been exposed to antibiotics and to have elevated levels of antibiotic resistance or tolerance [31]. In some cases, a type strain was more resistant than the gut-isolated strain to one antibiotic and less resistant to another, which can be explained by the fitness cost of maintaining multiple resistance genes [32]. Mutations resulting in antibiotic resistance are commonly associated with a reduction in growth rate, although that is not always the case [33].

### Susceptibility of bacterial strains to plant extracts

Medicinal and toxic plants were chosen for assessment of antibacterial activity in this study, based on anecdotal evidence and, where available, scientific literature. Interestingly, there is little in the way of systematic studies to assess whether plants used in traditional medicine are more likely to contain valuable bioactive compounds [34]. The use of certain plants in traditional medicine is not indicative of their efficacy; such data can only be provided when standardised and chemically-characterised plant preparations are tested in vitro and in animal models, and their efficacy is confirmed in clinical studies [35].

It was hypothesised that the type strains would be more susceptible to the plant extracts than the strains from the insect gut that have been exposed to the constituents of the extracts. Surprisingly this was not always true. Many of the gut-isolated strains that exhibited unexpected plant extract susceptibility also had some unusual differences in antibiotic resistance levels. For example, *Burkholderia fungorum* identified in the guts of the Cinnabar moth larvae feeding on ragwort was more susceptible to ciprofloxacin and kanamycin than the type strain. Apart from the elevated antibiotic resistance, the *Bu. fungorum* type strain was also more resistant to ragwort leaf extract. Similarly, the *Ba. vietnamensis* type strain was more resistant than the *Ba. vietnamensis* strain isolated from the guts of Beet Armyworms to ampicillin, chloramphenicol, ciprofloxacin, kanamycin and rifampicin, as well as periwinkle root extract. It is possible that the unexpected antibiotic resistance and plant extract susceptibility are correlated or linked, but it is difficult to rationalise the nature of this phenomenon, but it suggests that the interplay of bacterial strains in the insect gut is likely to be quite complex. For example, it is possible for toxic plant metabolites to be broken down in the gut before they are exposed to gut bacteria [36, 37].

### Isolation of natural products from plant extracts

A significant issue with this work was the difficulties experienced in isolating natural products from plant extracts. Initially crude extracts were fractionated using normal-phase HPLC, but the performance was unsatisfactory as the waxy components were binding too tightly to the silica gel stationary phase, requiring long wash cycles. To improve the HPLC efficiency, the plant extracts were partitioned between organic solvents and treated with SPE before fractionation. SPE is not as efficient in separating compounds as HPLC, but the variety of matrices available is larger. Two- or three-dimensional fractionation is possible, for example using ion-exchange matrices, size exclusion and silica- or polymer-based matrices that separate compounds based on their hydrophobicity. In the case of the plant extracts tested, size-exclusion chromatography produced peaks that were too broad and ion-exchange matrices introduced formic acid and ammonium hydroxide, which change sample pH and prevent the growth of bacteria. All experiments including ion exchange SPE included pH controls to avoid false positive results.

The HPLC fractionation was performed on a C18 matrix, which is normally used to separate mixtures of mostly hydrophilic to moderately hydrophobic compounds. Many plant extract components with antibacterial activity were not sufficiently retained by the C18 stationary phase, suggesting that hydrophilic interaction chromatography, ion-pairing reverse-phase or even ion chromatography may be required to separate these highly polar compounds. Additionally, multiple injections had to be made to obtain enough fractionated plant extract for antibacterial activity testing. The efficiency of HPLC decreases when more extract is loaded onto the column. Multiple small injections are favoured if the efficiency of the fractionation is required, making preparative HPLC a slow technique.

Our exploration of the insect/plant pairs suggests that it is straightforward to identify antibiotic resistance in the insect gut associated bacteria and to identify plant extracts with antibiotic activity. Isolation of bioactives from complex natural product extracts in quantities sufficient for structure elucidation, however, remains a challenging task.

### Antibacterial activity of the plant extracts

We have identified antibacterial activity in all plant extracts investigated, apart from the cabbage extract. As discussed before, it is not known which plants are more likely to have medicinal properties, making it difficult to hypothesise about the significance of this finding.

We only identified a mild antibacterial activity in the eucalyptus leaf extract. Antibiotic activity in eucalyptus extracts is normally described in the context of the eucalyptus essential oil. Eucalyptus oil (from *E. globulus*) is known to have an antibacterial activity, but it is relatively low compared to other essential oils [38]. Essential oils are notorious for their high content of reactive and unstable compounds. However, our plant extract processing methodology focused on and selected for hydrophilic compounds, as hydrophobic oily substances interfered with preliminary HPLC fractionations.

The cabbage extract was the only plant extract tested that had no antibiotic activity when tested against the bacteria from the Diamondback moth larvae guts and corresponding type strains. Very few studies describe antibacterial activity in cabbage, and those that do only find weak activity [39], suggesting that it is rooted only in anecdotal evidence of traditional medicine.

Ragwort contains high concentrations of pyrrolizidine alkaloids, which are responsible for its toxicity to cattle and horses. Because of the toxicity of pyrrolizidine alkaloids to generalist herbivores, the compounds are thought to be a feeding deterrent, however it was also suggested they protect the plants against microbial attack [40]. In our experiments ragwort extract was one of the most antibacterial plant extracts tested against insect gut bacteria, but the pyrrolizidine alkaloids were not detected in the plant extract. There are no data about antibacterial activities of pyrrolizidine alkaloids, but they have a moderate antifungal activity against plant pathogens [41]. These data suggest that pyrrolizidine alkaloids are not the compounds responsible for the antibiotic activity in the ragwort extract.

Unusually many HPLC fractions of the lavender extract were active against the bacteria tested. Individually each fraction was weakly active and together they contributed to the mild antibiotic activity of the plant extract. Weakly active lavender extract is consistent with mild evolutionary pressure on the rosemary beetle gut bacteria to develop resistance mechanisms. These data are not consistent with the antimicrobial activity of lavender essential oil. Lavender essential oil is a potent antimicrobial, active against both bacteria and fungi, but there is no consensus on which component of the essential oil is responsible for the activity [42]. Even though the scientific evidence indicates lavender has antibacterial properties, problems around standardization of the oil and the purification of the active components hamper the development of lavender preparations into a therapeutically useful agent.

The potato leaf extract was weakly antibiotic. Potato leaves, similarly to ragwort, are toxic to many herbivores, but they lack the strong antibacterial activity of ragwort extract. Potato leaves have not been previously investigated for antibacterial activity, as other potato waste products have been. Potato tubers unsuitable for sale have been investigated as a source of antibacterial activity [43], and had a mild level of antibiotic properties which have been attributed to phenolic compounds in the extracts. In summary, potato waste products are an attractive source to investigate potential bioactivity. However we have shown the level of antibiotic activity is relatively low and we have not pursued the purification of the active fraction of the extract.

### Antibiotic activity of the Madagascar periwinkle metabolites

Madagascar Periwinkle is a widely-studied plant in terms of its secondary metabolites. It has been found to produce terpenoid indole alkaloids that are used in cancer therapy [44], but its antibacterial activity is less well-documented. Extracts of leaves, stems, flowers and roots have been demonstrated to have antibacterial activity [45] and some of the main alkaloids present in Madagascar Periwinkle have been investigated for antibiotic activity [16]. The most active alkaloid found previously from this plant species was vindoline, but the MIC established for it was much higher than in our study. Additionally, Madagascar Periwinkle seeds contain antibacterial proteins CRCI and CRCII (*C. roseus* cystatin I and II) [46]. These proteins are thiol protease inhibitors and exhibit antibacterial activity against *Es. coli* and *Staphylococcus aureus*, but were ineffective against *Ba. subtilis*. At 25 μg/mL the zones of inhibition were 11–14 mm in diameter. The authors hypothesise that the cystatins are at least partially responsible for the medicinal properties of Madagascar Periwinkle in traditional preparations.

Our investigation is the first attempt to link the antibacterial properties of the Madagascar Periwinkle extract to the metabolites present in the plant. We have discovered that the main indole alkaloids have mild antibiotic activity against a wide range of bacteria. Vindoline had the highest antibacterial activity, especially when tested against a strain with a permeable cell membrane (Table 6). In general the compounds had MIC values an order of magnitude higher than most commonly used antibiotics, but they would be worth investigating further to determine their targets and modes of action. Indole alkaloids from Madagascar Periwinkle are already well-studied and that body of knowledge makes them attractive potential leads in antibiotic discovery.

## Conclusions

Using six exemplar insect/plant pairs we have shown that insect guts contain both environmental bacterial strains and strains typically associated with humans. In general, we found only a small number of culturable bacterial strains (2–6) from the insect guts, but a metagenomic analysis of the Giant Lime Green Stick insect gut showed that a larger number of species are likely to be present (at least 50–100). There has not been extensive metagenomic analyses of insect gut microbiota, but it appears that insect guts generally contain relatively few microbial species compared with mammalian guts [47]. We demonstrated that bacteria that could be cultured from the insect guts can be antibiotic-resistant (compared to their type strains from culture collections), as had been predicted from literature observations [6]. However, we also found that some species showed no difference in susceptibility to antibiotics between the gut-isolated and the type strains; this was not unexpected. More surprisingly we found several instances where the type strain was more resistant to antibiotics than the gut-isolated strain. In some cases, this can be rationalised by the fact that the origin of the type strain was a clinical isolate, but in other cases such rationalisation was not possible. Taken together these data suggest that comparing antibiotic susceptibilities in gut-isolated and type strain is not necessarily a reliable comparison and that the differences in the antibiotic resistance between gut-isolated strains and type strains cannot be used as a reliable indication of antibacterial activity in the food plant.

We confirmed the extracts of several of the plant species used have antibiotic activity, but that some plants, such as cabbage, lack obvious antibacterial activity. The susceptibility of the bacterial strains to the plant extracts can be used as a guide for identifying plant extract fractions with antibiotic activity. It was possible to fractionate the plant extracts and to identify fractions with antibacterial activity. These fractions were shown to be multi-component mixtures and further separation was not attempted. However, in the case of Madagascar Periwinkle, we could identify compounds in both the root and leaf extract with antibiotic activity and we established the minimal inhibitory concentrations of these and related compounds. Even though these metabolites only exhibit a relatively modest antibacterial activity, they are potentially interesting candidate compounds to follow.

## Methods

### Workflow

For each plant-insect pair the same work flow was followed. First the insect gut bacteria were identified and their antibiotic susceptibility profile was assessed. Then we tested the gut bacteria for susceptibility to the plant extract. Both the antibiotic susceptibility and plant extract susceptibility of the gut bacteria were compared between strains isolated from the insect guts and corresponding type strains obtained from culture collections. Our interest was in culturable strains, so that we could test them for susceptibility. However, in one case (*Diapherodes gigantea*) we used a culture-independent method to assess the variety of bacterial strains present in the gut microbiota (see below).

### Insects

Three adult Giant Lime Green Stick insects (*Diapherodes gigantea*) were provided by the John Innes Centre (JIC) insectary after they had died of natural causes. The insects were immediately frozen and stored at −20 °C until dissection. Five Diamondback moth (*Plutella xylostella*) larvae, ten Death’s-head Hawkmoth (*Acherontia atropos*) larvae and ten Beet Armyworm (*Spodoptera exigua*) larvae were obtained from the JIC Insectary. Eleven Cinnabar moth (*Tyria jacobaeae*) larvae were collected from Ragwort that was growing wild on the playing fields of University of East Anglia (Norwich, UK). Ten Rosemary beetles (*Chrysolina americana*) were collected from lavender plants on the JIC site. Where possible the insects were starved for two hours before dissection to enrich the gut contents in bacteria. The insects were flash frozen in liquid nitrogen and surface sterilized in 70% ethanol with a subsequent rinse in distilled water.

### Insect dissection

#### Rosemary beetle (*Chrysolina americana*) gut sample preparation

The rigid exoskeleton of the beetles prevented accurate dissection of the gut contents. Instead whole insects were homogenized with sterile micropestles in 1.5 mL Eppendorf tubes with 200 μL PBS buffer (8.0 g/L NaCl, 0.2 g/L KCl, 1.44 g/L Na_2_HPO_4_ and 0.24 g/L KH_2_PO_4_).

#### Other insect dissections

All steps of the dissection procedure were carried out in a biological safety cabinet. A sterile Petri dish was used as a dissecting surface. Using a sterile razor and stabilising the insect using sterile forceps the head of the insect was removed. Still stabilizing the insect body, an incision was made on the ventral side starting at the previous cut and running down the length of the body. The gut was identified as a large tubular structure running along the body. Where possible the gut tissue was cut open and the frozen gut contents were collected. In other cases the entire gut was removed. Gut contents were suspended in 200 μL PBS buffer and diluted 10, 100 and 1000 times. 50 μL of each dilution was plated on LB (LMM0202, Formedium; final concentrations: 1% (*w*/*v*) tryptone, 0.05% (w/v) yeast extract, 1% (w/v) NaCl), pH adjusted to 7.0 with HCl), LBG (LB medium plus glucose at 20 g/L (G8270, Sigma Chemicals)) and TSA (17.0 g/L peptone from casein, 3.0 g/L peptone from soymeal, 2.5 g/L glucose, 5.0 g/L NaCl, 2.5 g/L K_2_HPO_4_, pH adjusted to 7.3 with HCl), media (1% agar (AGA03, Formedium)). Plates were incubated for one to three days at 30 °C in aerobic conditions. Plates was also incubated using oxygen-absorbing pouches and sealed bags using Anaerocult (Merck) to generate anaerobic conditions. Isolates were streaked out on fresh agar plates and incubated as before. This ensured the purity of the bacterial isolates before identification.

### Identification of insect gut bacteria

Each collected insect was dissected and had its gut bacteria cultivated. We mainly used culture-dependent techniques for characterising the insect gut microbiota, in order to obtain strains that could subsequently be used in antibacterial activity testing of the plant extracts. However, in one case (*Diapherodes gigantea*) we used a culture-independent method to assess the variety of bacterial strains present in the gut microbiota (see below). For a summary of all isolated bacterial species, see Table 1. Each isolate was identified using 16S PCR with alkaline PEG reagent using 63f and 1389r primers (5′-CAGGCCTAACACATGCAAGTG-3′ and 5′-ACGGGCGGTGTGTACAAG-3′) and *Taq* DNA polymerase (28,104, Qiagen). Single colonies were picked from agar plates and resuspended in 500 μl distilled water and centrifuged at 6000 rpm for 4 min. Without disturbing the pellets, 490 μl sample was removed and 100 μl alkaline PEG reagent was added. The samples were mixed well by pipetting and incubated for 15 min at room temperature. 1 μl was added to the *Taq* PCR mix, prepared according to the manufacturer’s instructions. Reactions were carried out in a PTC-200 Thermo Cycler (MJ Research). The initial denaturation was carried out for 10 min at 95 °C, followed by 30 cycles of denaturation (95 °C for 1 min), annealing (57 °C for 1 min), and extension (72 °C for 2 min). The final extension was carried out at 72 °C for 10 min. The PCR products were soaked at 10 °C until further use. PCR products were separated on 1% agarose TAE (40 mM Tris, 20 mM acetic acid, 1 mM EDTA, pH 8.0) gels and purified using QIAquick PCR Purification Kit (28,104, Qiagen). DNA was sequenced using a BigDye v3.1 kit (Applied Bioscience) in a 10 μL reaction volume. Reactions contained BigDye 3.1 mix, 1× reaction buffer, 50–100 ng DNA template and 20 μM sequencing primer. Reactions were carried out in a PTC-200 Thermo Cycler (MJ Research). The initial denaturation was carried out for 1 min at 95 °C, followed by 30 cycles of denaturation (95 °C for 30 s), annealing (45 °C for 15 s), and extension (60 °C for 4 min). The final extension was carried out at 72 °C for 10 min. PCR products were soaked at 10 °C until further use. Samples were sent to The Genome Analysis Centre (Norwich, UK) for processing. Sequencing data was returned in the form of .txt and.abi chromatogram trace files. The sequences were trimmed to remove poorly recognized bases and run through the blastn algorithm (http://blast.ncbi.nlm.nih.gov/Blast.cgi) against “Nucleotide collection (nr/nt)” database.Bacteria were identified if the sequence was ≥97% similar to 16S RNA gene in the database and had an e-value close or equal to 0.

### Metagenomics

#### gDNA isolation

The genomic DNA (gDNA) was isolated from the lysed larval gut samples using FastDNA SPIN Kit for Soil (MP Biomedicals). 50 μL larval gut sample was added to the lysing matrix tube with 978 μL PBS buffer and 122 μL MT buffer (MP Biomedicals). The samples were homogenized in FastPrep instrument for 3 min at setting 6. The tubes were then centrifuged at 13,000 rpm for 15 min to pellet the cell wall debris. The supernatant was transferred to clean 2.0 mL microcentrifuge tubes and 250 μL Protein Precipitation Solution was added. The solutions were mixed by shaking the tube by hand 10 times. The samples were centrifuged at 13,000 rpm for 10 min to pellet the precipitated proteins. The supernatant was transferred to 15 mL tubes and mixed with 1 mL resuspended Binding Matrix. The tubes were inverted by hand for 2 min to allow binding of DNA and then placed in a rack for 3 min to allow settling of the silica matrix. 500 μL of supernatant was discarded without disturbing the settled Binding Matrix. The settled Binding Matrix was resuspended in the remaining supernatant and transferred to the SPIN™ Filter tubes. The SPIN™ Filter tubes were centrifuged at 13,000 rpm for 1 min. 500 μL SEWS-M buffer was added to the filter tubes and the Binding Matrix was resuspended gently, before centrifugation at 13,000 rpm for 1 min. The centrifugation was repeated to dry the filters of residual wash solution. The spin filters were dried for 10 min at room temperature and for 5 min at 37 °C. The Binding Matrix was resuspended in 100 μL DNase/Pyrogen-Free Water and centrifuged at 13,000 rpm for 1 min to elute the DNA. The samples were separated by electrophoresis on 1% agarose TAE gel to confirm the presence of large gDNA fragments.

#### Sequencing

The gDNA isolated from the stick insect guts was sequenced at ChunLab (Seoul, South Korea) using the MiSeq Nano platform. The filtered sequences were assembled into contigs, which were then classified into operational taxonomic units based on sequence similarity between them. Taxonomic classification was assigned to each operational taxonomic unit at the species level using the ChunLab’s EzTaxon-e database and blastn algorithm [48]. Chimeric sequences, which are contaminants originating from two separate DNA sequences, were filtered out using UCHIME program [49]. The sequencing reads were supplied in .clc format. The sequencing results analysed and visualised with CLcommunity software supplied by ChunLab. All sequence data were uploaded to EMBL-EBI.

### Other microbiological methods

#### Type strains

For each identified gut-isolated strain a corresponding type strain was obtained from one of the following culture collections: Centre de Resources Biologiques de l’Institut Pasteur (Paris), Health Protection Agency (Salisbury) or Deutsche Sammlung von Mikroorganismen und Zellkulturen (Brunswick). The strains obtained were: *Bacillus amyloliquefaciens* ATCC 23350, *Bacillus aquimaris* DSM 16205, *Bacillus licheniformis* ATCC 14580, *Bacillus vietnamensis* DSM 18898, *Burkholderia fungorum* CIP 107096 T, *Enterobacter asburiae* ATCC 35953, *Kocuria rhizophila* ATCC BAA-50, *Microbacterium foliorum* DSM 12966, *Microbacterium gubbeenense* DSM 15944, *Microbacterium oxydans* DSM 20578, *Microbacterium paraoxydans* DSM 15019, *Pantoea agglomerans* ATCC 27155, *Pseudomonas putida* ATCC 12633, *Raoultella terrigena* ATCC 33257, *Rhizobium pusense* DSM 22668, *Rhodococcus erythropolis* ATCC 4277, *Sanguibacter keddieii* ATCC 51767, *Sphingobacterium multivorum* ATCC 33613, *Staphylococcus epidermidis* ATCC 14990, and *Staphylococcus warneri* ATCC 27836; the origin of each strain is given in (Additional file 3: Table S1).

### Antibiotic susceptibility testing

#### Assay

The minimal inhibitory concentrations (MIC) for ampicillin, chloramphenicol, ciprofloxacin, kanamycin, rifampicin and tetracycline were determined by broth microdilution [50] for each gut-isolated strain and type strain pair. This panel of antibiotics was chosen based on their varied mechanisms of action [51]. Ampicillin inhibits the final stage of cell wall synthesis leading to cell lysis. Chloramphenicol prevents protein synthesis by inhibiting the peptidyl transferase activity of the ribosome. Ciprofloxacin is a gyrase inhibitor and kills cells by creating breaks in DNA. Kanamycin inhibits protein synthesis by binding to the 30S ribosomal subunit. Rifampicin disrupts RNA synthesis by inhibiting RNA polymerase. Tetracycline prevents protein synthesis by blocking the attachment of the aminoacyl-tRNAs to the ribosome.

To assess the levels of antibiotic resistance in the insect guts, we compared the antibiotic susceptibility profiles of the insect-gut isolated strains to type strains available from culture collections. Type strains were obtained from the CRBIP. HPA and DSMZ culture collections (Additional file 3: Table S1); a summary of the antibiotic susceptibilities is shown in Table 2. Briefly, 96-well plates with serial dilutions of antibiotics were inoculated with bacterial suspension and incubated for 24 h at 30 °C. The MIC was assigned when instead of a suspension of bacterial growth, a well with a clear broth was present. To confirm lack of bacterial growth, the OD was measured at 600 nm in a CLARIOstar plate reader (BMG Labtech). The control organism was *Es. coli* ATCC 25922. The MICs for vindoline, loganin, loganic acid, ajmalicine and catharanthine were also determined by broth microdilution for the gut-isolated strains from the beet armyworm, matching type strains, *Es. coli* (ATCC 25922), *Mycobacterium smegmatis* (ATCC 700084), *Ps. aeruginosa* (ATCC 15692) and *St. aureus* (ATCC 29213). 96-well plates with two-fold dilutions of the compounds were inoculated with bacterial suspension at OD_600_ = 0.08–0.11 and incubated for 24 h at 30 °C. The MICs were assigned as described above.

### Plant extract methods

#### Extract preparation

Plant extracts were prepared by homogenizing dried leaves of eucalyptus (*Eucalyptus dalrympleana*), cabbage leaves (*Brassica rapa*), lavender leaves and flowers (*Lavendula angustifolia*), ragwort leaves (*Jacobaea vulgaris*), potato leaves (*Solanum tuberosum*), and Madagascar periwinkle leaves and roots (*Catharanthus roseus*) with methanol. 100 g dried plant material was ground to fine powder either with a pestle and mortar or using an electric coffee grinder (Andrew James Stainless Steel Wet and Dry Coffee, Nut and Spice Grinder). The plant powders were soaked overnight in 300 ml methanol, filtered and soaked again twice in 300 ml methanol. The plant extracts initially had a deep green, nearly opaque colour and were soaked until they were no longer green. The three methanolic extracts were filtered, pooled and concentrated in an EZ-2 Elite evaporator (GeneVac). The extracts were de-fatted by liquid-liquid fractionation with petroleum ether in a 1:1 ratio. Only the methanol fraction was used in subsequent purifications. The crude eucalyptus extract was concentrated and de-fatted with petroleum ether. The aqueous partition was further purified using weak anion exchange solid phase extraction cartridges. The active methanol fraction was further fractionated by HPLC using a C18 reverse-phase column.

#### Antibacterial activity testing

To assess the antibacterial properties of the plant extracts, we tested the susceptibility of gut-isolated strains and type strains to these extracts. The plant extracts were prepared by drying the plant material in an evaporator (DNA SpeedVac, Savant), grinding it to powder and infusing in methanol at room temperature. The extracts were then filtered and crudely purified by solvent-solvent partitioning with petroleum ether.

It should be noted that to compare the antibacterial activity of extracts from different plants, the extracts were standardized. Each extract was dried until a dry pellet was obtained, which was resuspended in methanol (100 μg/mL). (Sometimes waxy material was present in the samples and the pellets would not appear dry. In such cases the pellet was evaporated until no more reduction in volume occurred and then weighed and resuspended in methanol at 100 μg/mL.) Resuspended extracts were used to infuse paper discs before assaying on bacteria from the insect guts and the corresponding type strains. This procedure was carried out to standardise the concentrations of plant extracts and compare their antibacterial properties.

Lawns of bacteria were prepared by overlaying TY agar plates with a mixture of 3 mL overnight bacterial culture at OD_600_ 0.08–0.11 and 3 mL molten and cooled TY agar. Paper discs (Whatman, 5 mm) were infiltrated ten times with 10 μL aliquots of the extract and placed on the bacterial lawns. After 24 h incubation at 30 °C the clear zones were measured.

### Activity-guided fractionation

#### Solid-phase extraction

Plant extracts with activity against bacteria were purified on solid-phase extraction (SPE) weak anion exchange columns (Oasis WAX 6 cm^3^ cartridge, Waters) according to the manufacturer’s instructions. Briefly, each column was primed with 6 mL methanol and calibrated with 6 mL 2% formic acid in MilliQ water (Merck) before loading no more than 5 mg sample resuspended in methanol. The columns were then washed with 6 mL 2% formic acid, 6 mL methanol and 6 mL 5% ammonium hydroxide in methanol. The flow through was collected, concentrated and assayed for antibiotic activity as described before.

#### High-pressure liquid chromatography

The active SPE fractions of plant extracts were subjected to reverse-phase high-pressure liquid chromatography (HPLC). Non-concentrated samples were used for the fractionation to avoid loss of separation when too much sample is loaded. The samples were centrifuged for 10 min. If required the supernatants were serially diluted at 1:10, 1:100 and 1:1000 ratios before injection. The samples were analysed on an HPLC instrument (Shimadzu Prominence/Nexera UHPLC). Separation was performed on an analytical 2 × 100 mm 3 μm Luna C18(2) column (Phenomenex) and semi-preparative 10 × 250 mm 5 μm Luna C18(2) column (Phenomenex), run at 0.3 mL/min for the analytical columns and 3 mL/min for the semi-preparative columns. All separations were run at 40 °C. A gradient of methanol against 0.1% formic acid in MilliQ water (Merck) was used to elute compounds. A general HPLC method consisted of a 10–100% methanol gradient over six column volumes followed by a 100% methanol wash over two column volumes and 10% methanol wash over two column volumes. UV/visible spectra (190–600 nm) and UV chromatograms (260 nm) were collected at 6.25 Hz with a time-constant of 0.16 s using the on-line detector between the HPLC column and the fraction collector. When fractions were collected, the fraction collector was set up to collect fractions of 2 mL throughout the duration of the method. After fractionation, the samples were dried in an evaporator and assayed as described before.

#### LC-MS analysis

Selected HPLC fractions were analysed on an LCMS-IT-ToF Mass Spectrometer. The samples used in the analysis were not concentrated. They were collected before the solvent was evaporated for antibiotic activity testing. The samples were mixed with 20% methanol in 1:4 ratio and centrifuged for 10 min. The supernatant was transferred to small glass inserts for analysis. Serial dilutions (1:10, 1:100 and 1:1000) of a sample were prepared when the original sample was too concentrated and reached above the limit of detection of the instrument. The samples were run on a Prominence/Nexera UHPLC system attached to an ion-trap ToF mass spectrometer (IT-ToF, Shimadzu). Separation was on a 100 × 2.1 mm 2.6 μm Kinetex EVO C18 column (Phenomenex) using a gradient of acetonitrile versus 0.1% formic acid in water, run at 0.6 mL/min at 40 °C. The gradient consisted of a 10–100% acetonitrile gradient over six column volumes followed by a 100% acetonitrile wash over two column volumes and 10% acetonitrile wash over two column volumes. Detection was by UV/visible absorbance and positive electrospray MS. UV/visible data were collected from 200 to 600 nm at 6.25 Hz with a time-constant of 0.16 s. Full MS data were collected from m/z 150–2000 with a maximum ion accumulation time of 20 ms, and automatic sensitivity control set to a target of 70% of optimum base peak intensity. The instrument also collected automatic MS2 data of the most abundant precursor ions at an isolation width of m/z 3.0, 50% collision energy, and 50% collision gas, and 15 ms ion accumulation time. The instrument was set up to collect two successive spectra of each precursor ion that was selected, and then ignore that precursor for 2 s in favour of the next most abundant precursor. Spray chamber conditions were 1.5 L/min nebulizer gas, 250 °C curved desorption line, 300 °C heat block, and drying gas “on”. The instrument was calibrated immediately before use, using sodium trifluoroacetate cluster ions according to the manufacturer’s instructions. The results were analysed with LabSolutions software (Shimadzu) and Profiling Solution software (Shimadzu).

## Additional files

Additional file 1: Figure S1. Rarefaction curves of metagenomic samples from two *D. gigantea* guts. (DOCX 240 kb)

Additional file 2: Figure S2. The composition of *D. gigantea* gut community. (DOCX 179 kb)

Additional file 3: Table S1. Sources of bacterial type strains. (DOCX 14 kb)

## Abbreviations

HPLC: high-performance liquid chromatography
LB: Luria broth
LC-MS: liquid chromatography-mass spectrometry
MIC: minimum inhibitory concentration
PBS: phosphate-buffered saline
PCR: polymerase chain reaction
PEG: polyethylene glycol
SPE: solid-phase extraction.
