# Supplementary material for: Antibiotic-resistant bacteria in the guts of insects feeding on plants: prospects for discovering plant-derived antibiotics
Source: BMC Microbiol. 2017 Dec 1;17:223. doi: 10.1186/s12866-017-1133-0 (PMC5709835; doi:10.1186/s12866-017-1133-0)
Supplement: Supplementary file 1 — Rarefaction curves of metagenomic samples from two D. gigantea guts. (DOCX 240 kb) [file 12866_2017_1133_MOESM1_ESM.docx]

**Figure S1**: **Rarefaction curves of metagenomic samples from two *D. gigantea* guts.** A rarefaction curve shows the rate of increase in the number of species that are discovered as more reads are being sequenced. These data indicate that one of the samples had larger species diversity as more OTUs were discovered from it and that both guts were sampled sufficiently to discover most strains present in them.
