# Supplementary material for: Antibiotic-resistant bacteria in the guts of insects feeding on plants: prospects for discovering plant-derived antibiotics
Source: BMC Microbiol. 2017 Dec 1;17:223. doi: 10.1186/s12866-017-1133-0 (PMC5709835; doi:10.1186/s12866-017-1133-0)
Supplement: Supplementary file 2 — The composition of D. gigantea gut community. (DOCX 179 kb) [file 12866_2017_1133_MOESM2_ESM.docx]

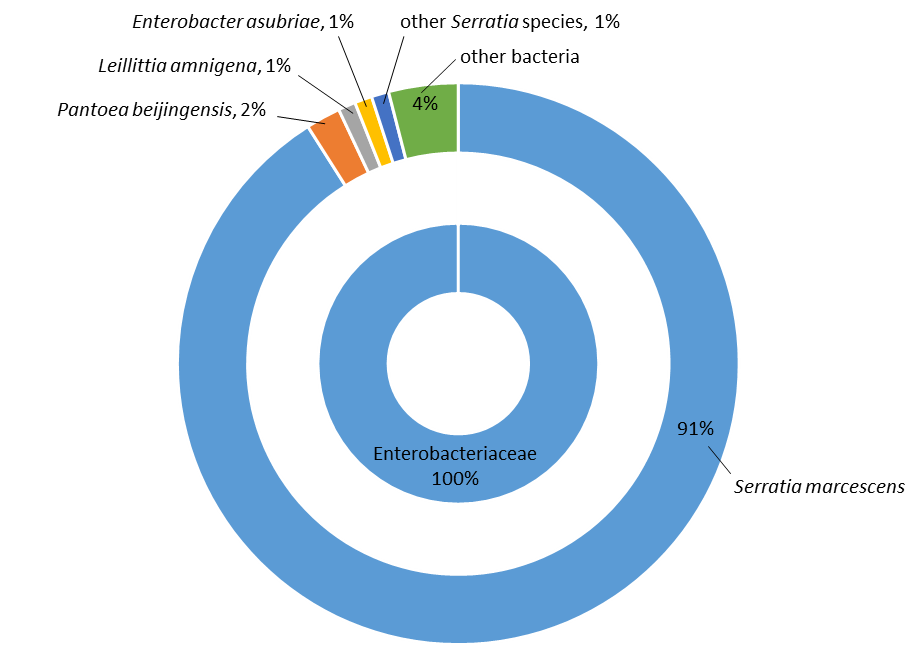

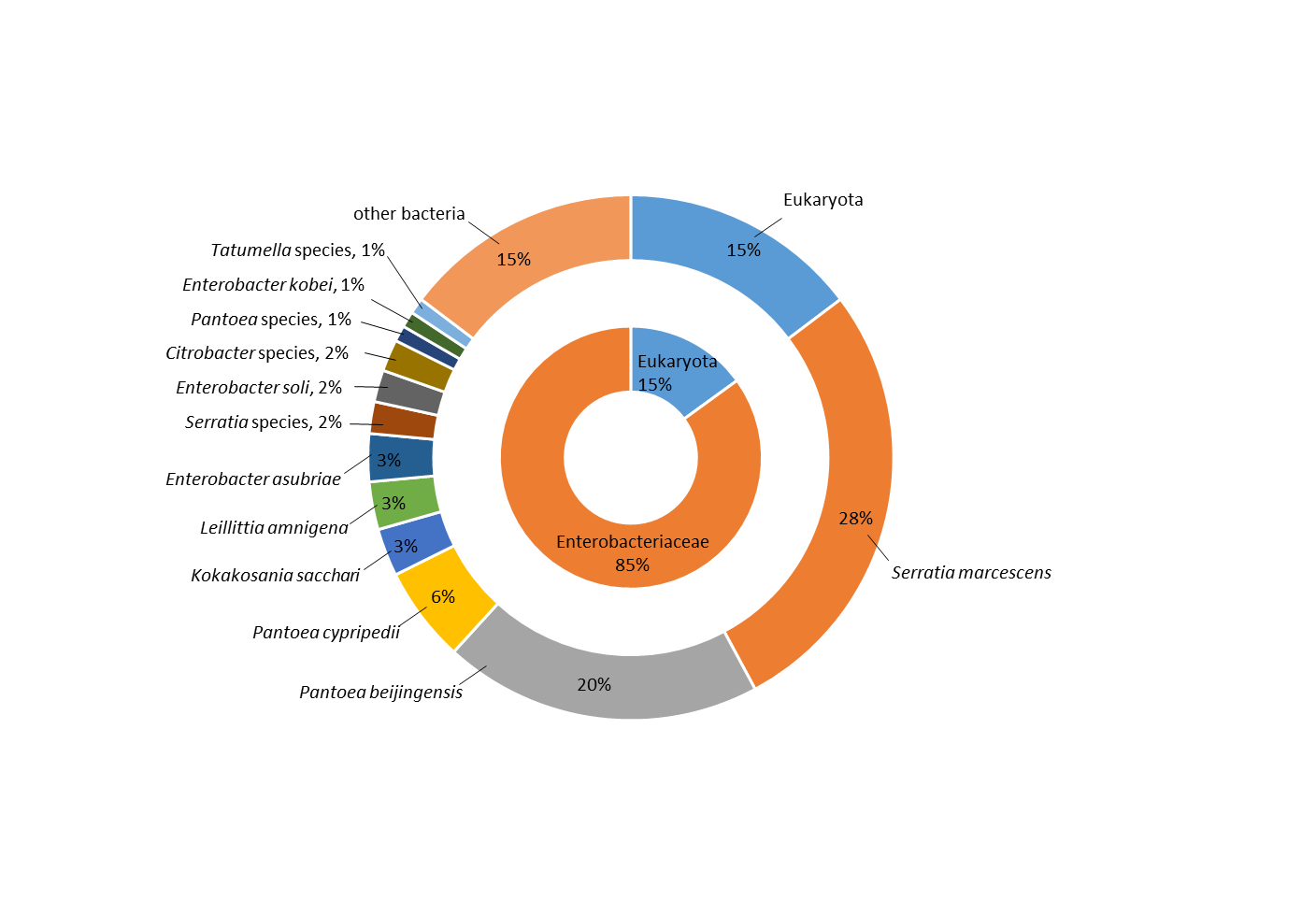
**Figure S2: The composition of *D. gigantea* gut community**. The composition is indicated by genus (inner ring) and species (outer ring); the two gut communities are shown separately. The first sample (a) had contaminant plant DNA and higher species diversity. The second community (b) was dominated by *S. marcescens*. Bacteria with less than 1% abundance were pooled together into the “other bacteria” category.

(b)

(a)
