# Supplementary material for: Antibiotic-resistant bacteria in the guts of insects feeding on plants: prospects for discovering plant-derived antibiotics
Source: BMC Microbiol. 2017 Dec 1;17:223. doi: 10.1186/s12866-017-1133-0 (PMC5709835; doi:10.1186/s12866-017-1133-0)
Supplement: Supplementary file 3 — Sources of bacterial type strains. (DOCX 14 kb) [file 12866_2017_1133_MOESM3_ESM.docx]

**Supplementary Table S1: Sources of bacterial type strains**

| **Species name** | **Collection number** | **Collection** | **Source** | **Pathogenicity group** | **Gram stain** |
| --- | --- | --- | --- | --- | --- |
| *Bacillus amyloliquefaciens* | ATCC 23350 | CRBIP | soil | 1 | positive |
| *Bacillus aquimaris* | DSM 16205 | DSMZ | sea water of a tidal flat | 1 | positive |
| *Bacillus licheniformis* | ATCC 14580 | DSMZ | soil | 1 | positive |
| *Bacillus subtilis* | ATCC 6051 | HPA | boiled hay infusion | 1 | positive |
| *Bacillus vietnamensis* | DSM 18898 | DSMZ | vietnamese fish sauce | 1 | positive |
| *Burkholderia fungorum* | CIP 107096T | CRBIP | fungus | 2 | negative |
| *Enterobacter amnigenus* | ATCC 33072 | HPA | soil | 2 | negative |
| *Enterobacter asburiae* | ATCC 35953 | DSMZ | lochia | 2 | negative |
| *Escherichia coli* | ATCC 25922 | HPA | human | 2 | negative |
| *Kocuria rhizophila* | ATCC BAA-50 | CRBIP | rhizosphere of *Typha angustifolia* | 1 | positive |
| *Microbacterium foliorum* | DSM 12966 | CRBIP | phyllosphere of grasses | 1 | positive |
| *Microbacterium gubbeenense* | DSM 15944 | DSMZ | surface of a smear-ripened cheese | 1 | positive |
| *Microbacterium oxydans* | DSM 20578 | CRBIP | air | 1 | positive |
| *Microbacterium paraoxydans* | DSM 15019 | DSMZ | human blood | 2 | positive |
| *Pantoea agglomerans* | ATCC 27155 | DSMZ | knee laceration | 2 | negative |
| *Pseudomonas putida* | ATCC 12633 | DSMZ | soil | 2 | negative |
| *Raoultella terrigena* | ATCC 33257 | CRBIP | drinking water | 1 | negative |
| *Rhizobium pusense* | DSM 22668 | DSMZ | rhizosphere of chickpea (*Cicer arietinum*) | 1 | negative |
| *Rhodococcus erythropolis* | ATCC 4277 | CRBIP | soil | 1 | positive |
| *Sanguibacter keddieii* | ATCC 51767 | CRBIP | bovine blood | 1 | positive |
| *Sphingobacterium multivorum* | ATCC 33613 | CRBIP | human spleen | 2 | negative |
| *Staphylococcus epidermidis* | ATCC 14990 | CRBIP | human nose | 2 | positive |
| *Staphylococcus warneri* | ATCC 27836 | CRBIP | human skin | 1 | positive |
